# Antidote to the Salts of Copper

**Published:** 1842-03

**Authors:** W. Benoist

**Affiliations:** Pharmaceutist at Sancoius (Chev)


*Antidote to the Salts of Copper.* By W. Benoist, Pharmaceutist at Sancoius (Chev).—Liquid albumen is at present almost exclusively adopted as an antidote for the salts of copper, since it possesses the property of decomposing and precipitating almost all the metals, and of forming albuminous compounds with the metallic oxide and the acid. But we are exposed to serious consequences in poisoning, if after having excited and facilitated emesis, we should administer to the patient a large amount of albumen, for the purpose of more certainly neutralizing the poison; what happens? The cupreous precipitate will be dissolved in the excess of albumen. If, on the contrary, too small a quantity of this latter is employed, it will remain dissolved in the excess of solution of copper. To obviate this inconvenience, we have no means, for we do not know what amount of albumen is necessary to neutralize a given quantity of any salt of copper and were this positively known, we could not properly estimate the quantity remaining in the stomach after emesis.

I am about to propose another agent which does not offer the same inconvenience; I mean a solution of carbonate of soda, which forms with the salts of copper precipitates of a green color, insoluble in water, carbonate of copper. It is on account of its insolubility in water, that this carbonate has no action on the animal economy. I have experimented on animals; some I caused to take acetate of copper, others the sulphate, and to others I gave at the same time both the salt of copper and alkaline solution. In every case the results were satisfactory. I caused a dog of moderate size to take 35 grains of a saturated solution of carbonate of soda. This quantity, more than sufficient to neutralize the poison, did not produce any unpleasant effects. I judge that this salt may be exhibited with impunity, in large amounts, as in the above cited case it only produced some alvine evacuations. The large quantity of carbonic acid which it contains (in 100 parts, carbonic acid 45, soda 31, water 29), causes it to be less caustic than the other alkaline carbonates. I have not yet had occasion to employ this in any case of poisoning in the human subject. I may observe that we ought not to lose sight of the indispensable necessity of exciting vomiting, so as to expel as much as possible the excess of poison.

Before M. Orfila had elevated toxicological science to its present rank, we may call to mind what were the means used to counteract the effects of the preparations of which I have spoken: solutions of sugar; these means being effectual, how can we explain the result? It may be that the carbon of sugar coming in contact with the oxygen of the water and of the oxide of copper, passes to the state of carbonic acid, and then the same reaction is produced as by the means I have recommended.

We have a recent example of the effect of sugar in an observation of M. Lesage, relative to a young man, who, wishing to destroy himself, took the sulphate of copper in a highly sweetened drink, and by accident escaped, the poison being thus neutralized.

From the foregoing, we may conclude that if sugar by one of its elements, may give rise to the formation of carbonic acid, and form insoluble compounds with the salts of copper, we should not be astonished that the bicarbonate of soda should possess this property in a higher degree.

Milk has also been for a long time considered an antidote to the salts of copper; the acid, it is true, separates the curd which precipitates; but it does not really render much service under these circumstances, unless it acts as a soothing application.—*American Journal of Pharm.* from *the Jour de Chimie Med.*

